# Deep Supervised Residual Dense Network for Underwater Image Enhancement

**DOI:** 10.3390/s21093289

**Published:** 2021-05-10

**Authors:** Yanling Han, Lihua Huang, Zhonghua Hong, Shouqi Cao, Yun Zhang, Jing Wang

**Affiliations:** College of Information, Shanghai Ocean University, Shanghai 201306, China; ylhan@shou.edu.cn (Y.H.); m190711295@st.shou.edu.cn (L.H.); zhhong@shou.edu.cn (Z.H.); y-zhang@shou.edu.cn (Y.Z.); wangjing@shou.edu.cn (J.W.)

**Keywords:** underwater image enhancement, details, residual, dense, deep supervision, GAN

## Abstract

Underwater images are important carriers and forms of underwater information, playing a vital role in exploring and utilizing marine resources. However, underwater images have characteristics of low contrast and blurred details because of the absorption and scattering of light. In recent years, deep learning has been widely used in underwater image enhancement and restoration because of its powerful feature learning capabilities, but there are still shortcomings in detailed enhancement. To address the problem, this paper proposes a deep supervised residual dense network (DS_RD_Net), which is used to better learn the mapping relationship between clear in-air images and synthetic underwater degraded images. DS_RD_Net first uses residual dense blocks to extract features to enhance feature utilization; then, it adds residual path blocks between the encoder and decoder to reduce the semantic differences between the low-level features and high-level features; finally, it employs a deep supervision mechanism to guide network training to improve gradient propagation. Experiments results (PSNR was 36.2, SSIM was 96.5%, and UCIQE was 0.53) demonstrated that the proposed method can fully retain the local details of the image while performing color restoration and defogging compared with other image enhancement methods, achieving good qualitative and quantitative effects.

## 1. Introduction

In recent years, marine information processing technology has developed vigorously, and the application of underwater object detection technology has become increasingly widespread. It involves the laying of submarine optical cables, the construction and maintenance of underwater oil platforms, the salvage of sunken submarine ships, the fishing of marine organisms, marine ecosystem research, and other fields. Underwater optical image has high resolution and abundant information with obvious advantages in short-distance underwater target detection. However, because of the influence of scattering and absorption of light, underwater images often have many quality problems such as noise interference, fuzzy texture features, low contrast, and color distortion. These lead to the lack of useful details in the target recognition of underwater images, bringing significant challenges to the marine fishery and other fields. Underwater image enhancement technology can effectively improve the quality of underwater images and provide powerful help for the realization of advanced tasks such as positioning and tracking of marine organisms. Therefore, in order for underwater images with high resolution and high color saturation to be obtained, it is of great significance to enhance the details of underwater images.

Compared with atmospheric optical imaging technology, underwater optical imaging technology is deeply affected by the absorption and scattering of light by water bodies. On the one hand, absorption is the energy loss caused by light reflected from an object entering the camera because water absorbs light of different wavelengths differently. Absorption can cause color attenuation. The energy absorption of red light is the largest, and that of blue-green light is the least, and thus underwater images show a blue-green tone. On the other hand, scattering is the energy loss caused by the change of direction of light in the transmission process caused by suspended particles or ambient light. In underwater imaging, the backscattering will cause background noise in the image and reduce the image contrast. The forward scattering causes the point light source to diffuse into a dispersion circle, resulting in fuzzy features such as textures, edges, colors, etc. Many underwater image processing technologies have emerged in order to solve the above problems, and they are mainly divided into image enhancement methods based on the non-physical model, image restoration methods based on the physical model, and image enhancement methods based on deep learning.

The non-physical model method mainly improves the visual effect by directly adjusting the pixel value of the image. For example, Iqbal et al. [1] proposed an unsupervised color correction method (UCM) for underwater image enhancement based on color balancing, contrast correction of RGB color model, and contrast correction of HSI color model. Orujov et al. [2] developed a contour detection-based image processing algorithm based on Mamdani fuzzy rules, which uses contrast-limited adaptive histogram equalization for contrast enhancement and median filter for background exclusion. Versaci et al. [3] presented a new fuzzy edge detector based on both fuzzy divergence and fuzzy entropy minimization for the threshold sub-step in gray-scale images. A new variational model [4] was proposed on the basis of total variation for simultaneously removing the tangling noise, estimating the location of missing pixels, and filling them in. An underwater image enhancement method [5] based on local contrast correction and multi-scale fusion was proposed to resolve low contrast and color distortion of underwater images. The non-physical model method is prone to result in color deviation and artifact, oversaturation, and undersaturation since it does not consider the optical characteristics of underwater imaging.

The physical model method is to build a mathematical model for the degradation process of underwater images. First, one estimates the parameters according to the model, and after that reverses the clear underwater image. For example, Drews et al. [6] (UDCP) proposed a method to estimate the transmission in underwater environments that consists of an adaptation of the dark channel prior, a statistical prior based on properties of images obtained in natural outdoor scenes. Liu et al. [7] developed an underwater image restoration method based on adaptive attenuation-curve prior that can simulate the light attenuation process in different underwater scenes and effectively eliminate the effects of image noise, haze, and artificial lighting, allowing for image de-blurring, color enhancement, and edge detail preservation. The physical model method is usually based on some prior assumptions, and thus it has certain limitations. Moreover, the parameter estimation algorithm is highly complex.

As is broadly known, deep learning has developed rapidly and is widely used in various computer vision and image processing tasks. Some studies have tried to use data-driven technologies (such as convolutional neural networks and generative adversarial networks) to cope with the problem of image improvement. WaterGAN [8] took in-air images and depth maps as input and generated corresponding synthetic underwater images using real unlabeled underwater images to learn a realistic representation of water column properties of a particular survey site. Then, these data served as input to a novel end-to-end network for color correction of monocular underwater images. Wang et al. [9] proposed an unsupervised generative adversarial network (UWGAN) for generating realistic underwater images from in-air image and depth map pairs. Then, the synthetic underwater datasets were used to train Unet for color restoration and defogging. Li et al. [10] proposed an underwater image enhancement model based on underwater scene priors. It directly reconstructed the clear latent underwater image while preserving the original structure and texture by jointly optimizing multi-term loss. Benefiting from the light-weight network design and effective training data, the model can be extended to underwater video for frame-by-frame enhancement. Islam et al. [11] presented a conditional generative adversarial network-based model (FunieGAN) for real-time underwater image enhancement. The model planned an objective function that evaluated the perceptual image quality on the basis of its global content, color, local texture, and style information, achieving favorable results in underwater target detection and saliency detection. Li et al. [12] developed a light enhancement net based on the convolutional neural network. It solved the problem of lack of detail in images of road scenes in low-light situations and reduced the risk of a crash risk of connected autonomous vehicles. The image enhancement methods based on deep learning can effectively improve the image quality compared with traditional algorithms, but there are some shortcomings in detailed enhancement because of the complexity of the underwater environment. The existing deep learning frameworks fail to retain and transfer the details of underwater images to the utmost extent and cannot fully extract the deeper features of underwater images, which lead to the color imbalance and details blurring of the restored images. Therefore, in order to further improve the quality of underwater images, researchers must design a method that can transfer more sufficient image information and extract more accurate image features to enhance underwater images.

**Figure 1:**
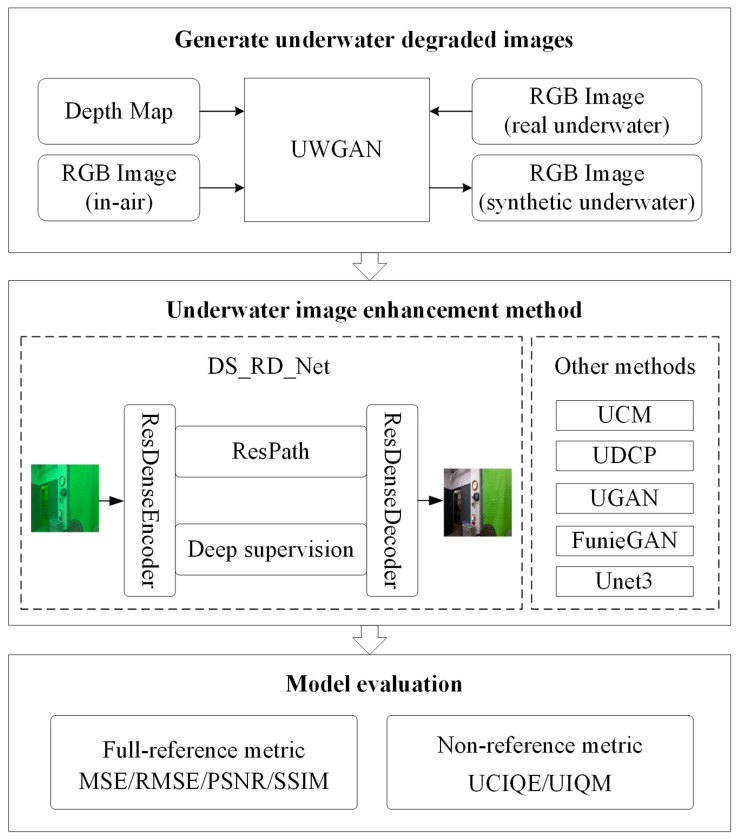
The proposed general framework for underwater image enhancement (three major parts: synthetic model of underwater images based on UWGAN, underwater image enhancement network based on DS_RD_Net, and evaluation of underwater image enhancement methods).

On the basis of the above research, this paper proposes a deep supervised residual dense network (DS_RD_Net) for underwater image enhancement, which trains an encoding-decoding network to learn the mapping relationship between clear in-air images and synthetic underwater degraded images. It fully preserves the detailed information, such as the content structure of the original image while restoring the color and removing the fog of the original image. Figure 1 shows the entire framework for the underwater image enhancement method based on a deep supervised residual dense network.

(1) Because it is difficult to obtain real clear underwater images, taking real underwater images, in-air images, and depth maps as input, we used UWGAN to generate a large training dataset of paired images by training generator and discriminator.
(2) DS_RD_Net is proposed for underwater image detailed enhancement. Residual dense blocks are adopted to fully extract features of different levels and different scales in order to reduce the loss of details during feature propagation. Residual path blocks are added to the skip connection between the encoder and decoder, which balances the semantic differences between the low-level features and high-level features and reduces the loss of details in the down-sampling process.
(3) A deep supervised mechanism is introduced to guide the DS_RD_Net training and improve the gradient propagation, thereby enhancing the robustness of the network and enabling the model to achieve a good enhancement effect on images with different degrees of degradation.
(4) We executed extensive performance comparisons between DS_RD_Net and other underwater image enhancement methods and demonstrated the effectiveness of the proposed method quantitatively and qualitatively.

The remainder of this paper is structured as follows. In Section 2, we briefly introduce the principle of underwater imaging and the synthesis method of degraded underwater images. In Section 3, the DS_RD_Net for underwater image enhancement is presented and analyzed. In Section 4, extensive experiments are conducted to demonstrate and discuss the effectiveness of the proposed method and the advantages over current methods. In Section 5, we conclude this paper.

## 2. Synthesis Algorithm of Underwater Images

### 2.1. The Imaging Principle of Underwater Images

**Figure 2:**
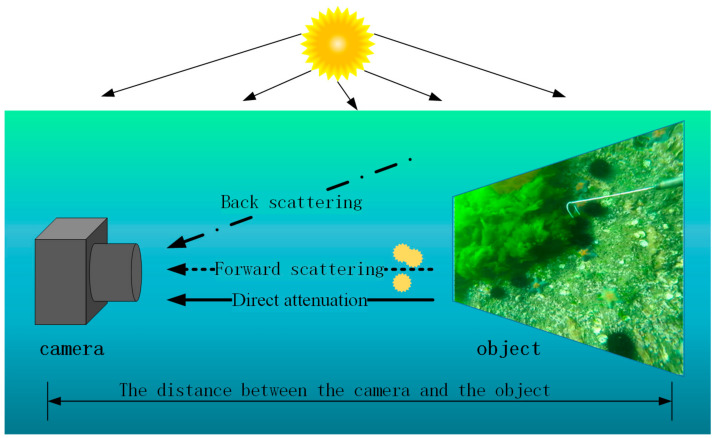
Physical model of underwater imaging.

When propagating in water, light is strongly attenuated by the scattering of particles and impurities and the absorption of the water medium. Scattering is a small-angle deflection phenomenon when light travels along a straight line. Absorption is the energy loss caused by light in the propagation process. According to Jaffe [13], as shown in Figure 2, the underwater image can be represented as the summation of three components (as shown in Equation (1)). The direct attenuation component represents the light that is reflected by underwater objects without being scattered. The forward scattering component means that the reflected light of the object is scattered at a small angle due to particles. The backscattering component refers to the reflected light of surrounding scattered by particles.

(1) $$E_{r}=E_{d}+E_{f}+E_{b}$$

where, $E_{r}$ is the light entering the camera, $E_{d}$ represents the direct attenuation component, $E_{f}$ represents the forward scattering component, and $E_{b}$ represents the backscattering component.

### 2.2. Synthetic Model of Underwater Images

In general, the typical characteristics of images taken in an underwater environment are as follows. One is the contrast reduction caused by backscattering and the details blurring caused by the forward scattering. The other is color attenuation caused by the different absorption of light by different wavelengths of water. The energy absorption of red light is the largest, and that of blue-green light is the least; thus, underwater images show a blue-green tone. Therefore, it is of great significance to study the imaging principle of underwater images, which can help us to better refine underwater images.

Deep learning has demonstrated brilliant success in modeling complex nonlinear systems. Still, it requires much training data that are difficult to obtain in deep-sea environments, and it is also difficult for underwater images to have corresponding label values. The generative adversarial network can generate a large training dataset of paired imagery, including synthetic underwater images and real in-air images. These data serve as input to a novel end-to-end network for color correction.

**Figure 3:**
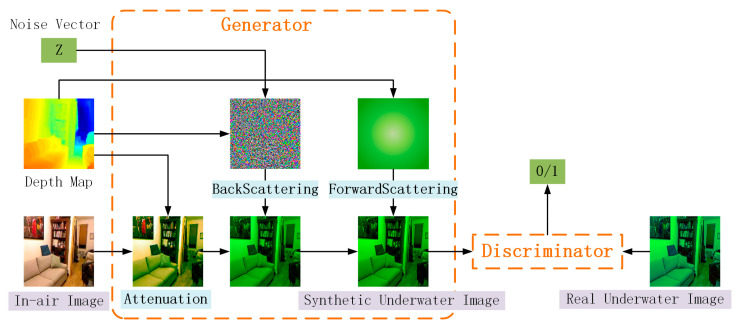
UWGAN architecture. UWGAN takes in-air images and its depth maps as input; then, it synthesizes underwater degraded images on the basis of underwater optical imaging model by generative adversarial training.

UWGAN [9], a generative adversarial network, taking in-air images and depth maps as input, synthesizes underwater degraded images by training generator and discriminator. The generator simulates the imaging process of underwater images, including three stages of direct attenuation, backscattering, and forward scattering, in order to output a synthetic image that the discriminator classifies as real. Using the synthetic underwater images and the real underwater image as input, the discriminator classifies each sample as real (1) or synthetic (0) to correctly classify the synthetic underwater images and the real underwater images. UWGAN network structure is shown in Figure 3.

## 3. Deep Supervised Residual Dense Network

**Figure 4:**
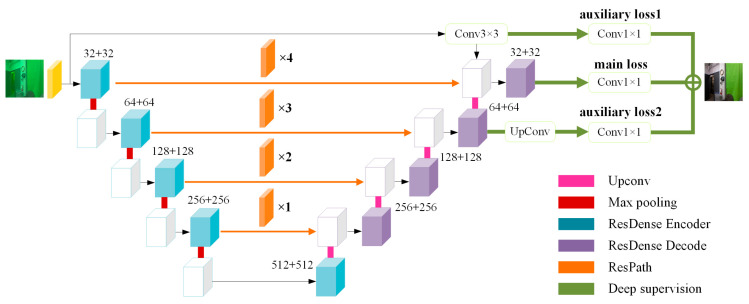
DS_RD_Net architecture. DS_RD_Net adds residual dense blocks, residual path blocks, and a deep supervision mechanism to learn the mapping relationship between clear in-air images and synthetic underwater degraded images.

In this paper, a deep supervised residual dense network for image enhancement is proposed to better learn the mapping relationship between the in-air images and the synthetic underwater degraded images. We designed the modules of feature extraction, feature fusion, and supervision mechanism to preserve the structure and content details of the original image as much as possible while restoring color and defogging so that the image will not be affected when used for advanced computer vision tasks (such as target detection, etc.). The overall network architecture is shown in Figure 4. We used residual dense blocks to extract features of different levels and scales fully. We then added residual path blocks to alleviate the semantic differences between encoder features and decoder features so that low-level features and high-level features were compatible with each other and effectively merged. After that, we introduced a deep supervision mechanism to guide the training process to improve gradient propagation.

### 3.1. Residual Dense Blocks

The absorption degree of water medium to the light of different wavelengths is different, leading to the color polarization of underwater images. Scattering caused by water impurities will reduce the image contrast. Distortion caused by scattering will blur image details. Therefore, this increases the difficulty of information extraction, making underwater images difficult to recognize. It is especially important to retain the detailed features of the original image when enhancing the image. The residual dense blocks can make full use of the features of different levels and scales to extract detailed features such as edges, textures, and colors as much as possible to reduce the loss of details during feature propagation.

**Figure 5:**
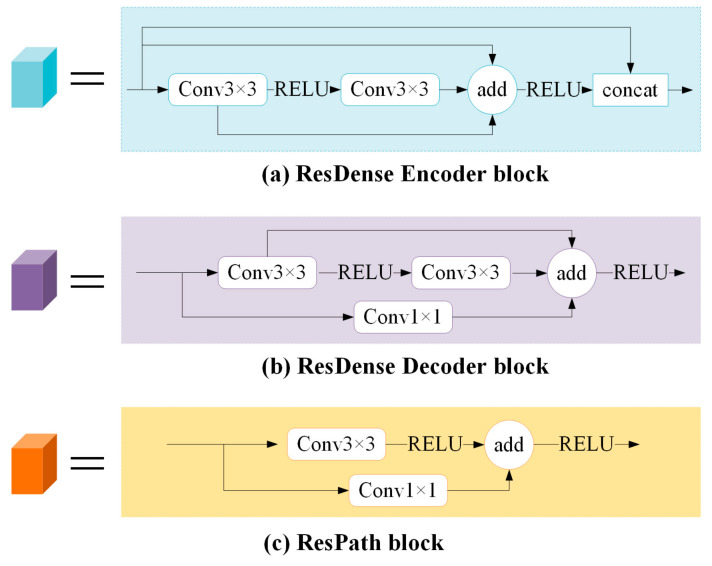
Details of several blocks: (**a**) residual dense encoder block; (**b**) residual dense decoder block; (**c**) residual path block.

In the paper, residual dense blocks [14] were used to extract features, as shown in Figure 5a,b.

- The encoder: Firstly, similar to ResNet [15], the ResDense Encoder block (Figure 5a) contains two convolution-ReLU operations and allows the original input to be directly passed to the back of the second convolution so that the information integrity can be protected only by learning the difference between the input and output. It can alleviate the loss in the process of feature extraction and improve the problems of gradient vanishing and gradient explosion. Secondly, a connection is added between the output of the first convolution and the output of the last convolution, which allows the parameters to be updated in the first convolution even if the gradient in the second convolution is close to zero. At this time, the feature aggregation operation enables rich information to be combined for feature extraction in consecutive layers, which is sufficient to back propagate the gradient effectively. Thirdly, like DenseNet [16], the input and output of two convolution-ReLU are superimposed on the dimension, realizing feature reuse and maximize the information flow in the network. This densely connected mode can obtain rich features through fewer convolutions.
- The decoder: The ResDense Decoder block (Figure 5b) also contains two convolution-ReLU operations and adds a connection between the output of the first convolution and the output of the last convolution. The difference is that the original input is not directly passed to the back of the second convolution, but conv1×1 is added to the connection. More nonlinearity is introduced to enhance the expressive ability of the network.

### 3.2. Residual Path

Underwater images are blurry and distorted in color. Aquaculture creatures such as holothurians, echinus, and scallops tend to be small and occupy a small pixel area in the images with blurred boundaries. Down-sampling can shrink the size of the feature map and reduce the number of parameters and the amount of calculation, but it will lose the spatial location and details of the target. In order to reduce the loss of details in the down-sampling, one needs to introduce the residual path blocks into the feature fusion module.

In the paper, residual path block [17] (Figure 5c) was added to the skip connection of Unet. The introduction of shortcut connections before the max-pooling and after the up-sampling enables the network to propagate the spatial information lost during the max-pooling. However, the encoder features are lower-level features, and the decoder features are higher-level features. There may be semantic differences in the direct fusion of two incompatible features. Therefore, some residual operations were added on the skip connection to make the two features reach the same depth before splicing in order to balance the semantic differences between low-level features and high-level features. In addition, the features of the encoder are processed by more convolutions as the network deepens, and therefore the number of differences will gradually decrease as the skip connection deepens. That is, 4, 3, 2, and 1 residual path blocks will be used in residual path 1, 2, 3, and 4 layers, respectively.

### 3.3. Deep Supervision

In most networks, only the results of the output layer are supervised, while the supervision of hidden layer features is ignored. Deep supervised mechanism [18] introduces a classifier to calculate classification errors in the hidden layer and takes the error calculated in the hidden layer as an auxiliary loss and the output error of the last layer of the network to form a joint objective function to guide network training. It is used to solve the problems of the vanishing gradient and the slow convergence of deep neural networks. In this paper, the deep supervision mechanism is shown in Figure 3. The input of the first branch is the output result of 7 × 7 and 3 × 3 convolutions of the input image. The first branch contains a 1 × 1 convolution, and the auxiliary loss function is auxiliary loss1. The input of the second branch is the output result before the last up-sampling of the decoder. The second branch contains an up-sampling and a 1 × 1 convolution, and the auxiliary loss function is auxiliary loss2. The output of the last layer of the network and the output of the two hidden layers are denoted as the backbone network output and branch network output, respectively. Deep supervision does not only directly supervise the output layer, but it also supervises the output layer and the hidden layers at the same time. In the procedure of minimizing the loss, the prediction results of the backbone network and the branch network are jointly employed to improve the gradient propagation.

The paper uses L1_L2 joint loss function as the loss function of the backbone network and the branch network. L1 loss is defined as Equation (2), L2 loss is defined as Equation (3), and L1_L2 joint loss is defined as Equation (4) [9].

(2) $$L^{l1}\left(X\right)=\frac{1}{N}\underset{x\in X}{\sum }\left|p\left(x\right)-g\left(x\right)\right|$$

(3) $$L^{l2}\left(X\right)=\frac{1}{N}\underset{x\in X}{\sum }{\left(p\left(x\right)-g\left(x\right)\right)}^{2}$$

(4) $$L^{l1\_l2}\left(X\right)=\alpha \cdot L^{l1}\left(X\right)+\left(1-\alpha \right)\cdot L^{l2}\left(X\right)$$

where X represents a collection of pixels in the image area, x represents a pixel point in the image area, $p\left(x\right)$ represents the pixel value of the reconstructed image, and $g\left(x\right)$ represents the pixel value of the reference image. α is a hyperparameter. On the basis of experience, we set the scale factor α to 0.8.

(5) $$main\;loss=auxiliary\;loss1=auxiliary\;loss2=L^{l1\_l2}\left(X\right)$$

(6) $$L\left(X\right)=\beta \cdot main\;loss+\gamma \times auxiliary\;loss1+\delta \times auxiliary\;loss2$$

where both the backbone network loss and the branch network loss adopt the L1_L2 joint loss function, as shown in Equation (5). In Equation (6), the main loss of the backbone network as well as the auxiliary loss1 and the auxiliary loss2 of the branch are given different weights by the scale factors β, γ, and δ, respectively. Finally, the three loss functions jointly guide the entire network training.

## 4. Experiments and Discussions

### 4.1. Dataset

The in-air datasets we used were images of indoor scenes that had been labeled in the NYU Depth dataset V1 [19] and the NYU Depth dataset V2 [20]. The labeled datasets were composed of pairs of RGB and depth frames that had been synchronized. The V1 datasets had 2284 images and 2284 depths, and the V2 datasets had 1449 images and 1449 depths. We mixed the V1 dataset and the V2 dataset into one dataset, including 3733 images and 3733 depths. The underwater datasets were the official training datasets provided by the 2020 Underwater Object Detection Algorithm Contest, including four categories: holothurian, echinus, scallop, and starfish. There were 594 images with 1920 × 1080 resolution, 1630 images with 3840 × 2160 resolution, 3237 images with 720 × 405 resolution, 44 images with 586 × 480 resolution, and 38 images with 704 × 576 resolution, a total of 5544 images. For convenience, 5544 training images were converted into 608 × 608-pixel images to meet the input data size that the model can receive.

### 4.2. Experimental Setup

The training settings of our proposed method are presented in detail in this section. Our models were trained in the computer with the following configurations: Intel i9-10920X processor, 64 GB RAM, GeForce RTX 2080Ti graphics card.

Firstly, UWGAN was trained to synthesize underwater degraded images using the labeled NYU Depth V1 and NYU Depth Dataset V2, and the 2020 Underwater Object Detection Algorithm Contest datasets. The model was trained for 30 epochs, using Adam optimizer with a learning rate of 0.0001, and the momentum term was set to 0.5. The batch size was set to 8 with output images set to 608 × 608. Secondly, DS_RD_Net was trained as an image enhancement network using synthetic pairs (including 3733 clear in-air images and 3733 synthetic underwater degraded images). The batch size was set to 2 and the output image size was 608 × 608. The learning rate was set to 0.0001 according to Adam optimizer, and the momentum term was set to 0.5. Our model was trained for 200 epochs.

### 4.3. Ablation Experiments and Analysis

To verify the effectiveness of our proposed method, we conducted five ablation experiments to evaluate the residual dense blocks, the residual path blocks, and the deep supervised mechanism. First, the Unet [9] (referred to as Unet3) was used as the baseline. Second, a down-sampling layer was added on the basis of Unet3 (referred to as Unet4). Third, on the basis of Unet4, we updated the ordinary convolutions into residual dense blocks (referred to as RD-Unet). Fourth, we added residual path blocks on the basis of RD-Unet (referred to as RD_RP-Unet). Finally, DS_RD_Net was proposed. On the basis of RD_RP-Unet, we added two auxiliary losses before the first down-sampling in the encoder and after the last up-sampling in the decoder, combined with the loss of backbone network to supervise the training of the network jointly (referred to as DS_RD_Net).

Four full-reference indicators, namely, MSE, RMSE, PSNR, and SSIM, were used to evaluate the image quality on synthetic underwater datasets. The higher the PSNR score, the lower the RMSE and MSE scores, indicating that the result is closer to the reference image in terms of image content. The higher the SSIM score, the closer the result is to the reference image with respect to the image structure and texture. Table 1 shows the average scores of 3733 synthetic underwater datasets. The following are detailed analyses of the image enhancement effects of several strategies.

Firstly, shallow networks can extract simple features such as edge, color, and texture of images, while deep networks can extract abstract features of images by more convolutions to obtain a larger receptive field. It can be seen from Table 1 that the SSIM of Unet4, by adding a down-sampling layer, was 2% higher than that of Unet3. MSE and RMSE, reduced by 44 and 3, respectively, and PSNR increased by about 4. This demonstrates that adding a down-sampling layer helps to extract the deeper semantic features of the image. Secondly, the propagation of features will gradually weaken, and the gradient will easily disappear as the network deepens. Comparing the results of the Unet4, we observed that the SSIM of RD-Unet was improved to 95.26% from 94.66%, the MSE and RMSE descended, and PSNR ascended. It proves that residual dense blocks can effectively improve the problem of gradient disappearance and enhance the propagation of features to improve the utilization of features. Thirdly, the features in the encoder were low-level features due to the shallower convolutional layers, while the features in the decoder were higher-level features due to the deeper convolutional layers. There were large semantic differences between them, and thus it was not appropriate to directly splice. Compared with RD-Unet, the MSE and RMSE of RD_RP-Unet decreased by 7 and 0.6, respectively, PSNR increased by 0.9, and SSIM rose from 95.26% to 96.05%. Therefore, adding residual path blocks before splicing can make them have a consistent depth to achieve an effective fusion of shallow features and deep features. Fourthly, DS_RD_Net had improved performance relative to RD_RP-Unet: MSE decreased from 23.07 to 19.35, RMSE reduced by 0.3, PSNR increased to 36.2, and SSIM increased to 96.47%. The results indicate that the deep supervision mechanism can improve the learning ability of the network by improving the learning ability of the hidden layer and reduce the information loss triggered by down-sampling and feature propagation.

In addition, we used two non-reference indicators, UCIQE [21] and UIQM [22], to evaluate the image quality on real underwater datasets. The higher the UCIQE score, the better the balance between chroma, saturation, and contrast; the higher the UIQM score, the better the result is in terms of being line with human visual perception. Table 2 shows the average scores of 5544 real underwater datasets.

As can be seen from Table 2, the DS_RD_Net improved the UCIQE score by 0.13 and improved the UIQM score by 0.6 in comparison with the original images. In addition, the combination of residual dense blocks and residual path blocks achieved the highest UCIQE score of 0.5376. This states that our method can balance the chroma saturation and contrast of underwater images to a certain extent, as well as being able to improve the quality of underwater images. However, neither of our strategies significantly improved the UCIQE score nor the UIQM score compared with Unet3 because the quantitative results of non-reference indicators largely depend on the value of the scale factor. On the contrary, our strategies are to keep the structure and content of the original image as much as possible without changing the value of the scale factor. Although some enhanced images can achieve higher scores, the visual quality is poor because the metric is calculated in pixels. Therefore, we believe that there is a certain gap between quantitative scores of non-reference indicators and subjective visual quality. In other words, the image quality evaluation indexes UCIQE/UIQM currently designed for underwater images have limitations in some cases [23].

### 4.4. Comparison Experiments and Analysis

In this section, we quantitatively and qualitatively compare our proposed method with several representative underwater image enhancement algorithms, including UCM [1], UDCP [6], UGAN [24], Unet3 [9], and FunieGAN [11].

#### 4.4.1. Qualitative Evaluations

**Figure 6:**
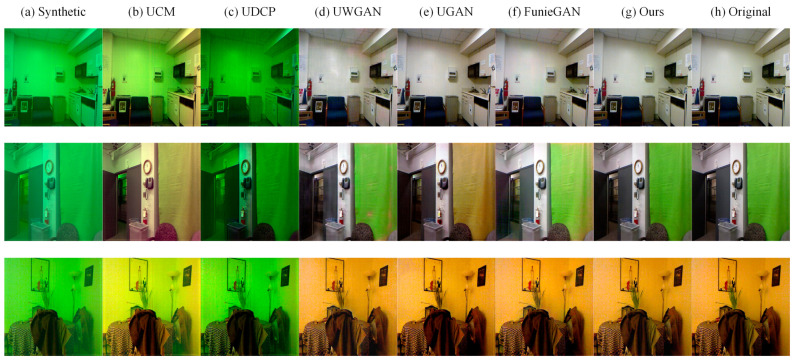
Qualitative comparisons for samples from synthetic underwater datasets. (**a**) Synthetic underwater degraded images. (**b**) Results of UCM. (**c**) Results of UDCP. (**d**) Results of Unet3. (**e**) Results of UGAN. (**f**) Results of FunieGAN. (**g**) Our results. (**h**) Ground truth.

**Figure 7:**
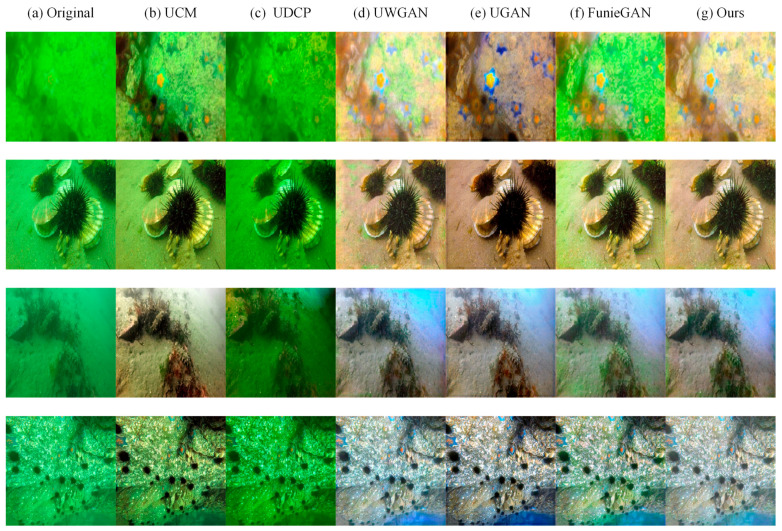
Qualitative comparisons for samples from real underwater datasets. (**a**) Real underwater images. (**b**) Results of UCM. (**c**) Results of UDCP. (**d**) Results of Unet3. (**e**) Results of UGAN. (**f**) Results of FunieGAN. (**g**) Our results.

Firstly, we compared the capability of different methods to improve the visibility of images on synthetic underwater datasets and real underwater datasets. Qualitative comparisons are shown in Figure 6 and Figure 7. UCM can enhance the brightness and contrast of the image, but it seems to be over-enhanced in some areas of the image. UDCP has a poor enhancement effect, and it darkens the image but enhances the contrast of the image. UGAN and FunieGAN can enhance the contrast of the image, but they do not recover color well and generate some artifacts, destroying the structure of the image. UGAN can restore the color of the image better but introduces some noise. The proposed method can restore the color of underwater images while maintaining the proper brightness and contrast, and almost no additional noise is introduced. Compared with other methods, the visual quality of our results is similar to the ground truth.

#### 4.4.2. Quantitative Evaluations

Full-reference indicators MSE, RMSE, PSNR, and SSIM were used to evaluate the performance of different methods on 3733 synthetic underwater datasets. The comparison results in Table 3 reveal that our proposed method achieved the best results in all aspects.

The MSE and RMSE of UCM and UDCP were too high, while the PSNR and SSIM were too low, indicating that these two methods can improve the image contrast but can also aggravate the influence of noise and reduce the detail of images. UGAN and FunieGAN use a generative adversarial network to learn global content, color, and local texture of images, as well as obtain smaller MSE and RMSE and larger PSNR, with SSIM reaching 95%. Unet3 adopts the idea of encoding and decoding to learn the mapping relationship between clear images and degraded images to enhance the degraded images. The PSNR was increased to 29.8 and the SSIM value reached 92%. Compared with other methods, our method obtained the minimum MSE of 19.3452, the minimum RMSE of 4.1534, the maximum PSNR of 36.2106, and the maximum SSIM of 96.47%. Experimental results illustrate that our method does not introduce too much noise in the process of image enhancement and preserves the details of image structure and content.

Table 4 shows average scores of UCIQE and UIQM of 5544 real underwater images and enhanced images based on different methods. Compared with the original image, both indicators of our method were improved to a certain extent, with an increase of 0.13 for UCIQE and 0.6 for UIQM. The experimental results show that the proposed method can effectively balance the hue, saturation, and contrast of underwater images and improve the visibility of images.

Among the six methods, our method ranked third in UCIQE scores, about 0.04 lower than the best-performing UCM, and fourth in UIQM scores, about 0.3 lower than the best-performing UGAN. The proposed method can retain the details of the structure and content of the original image as much as possible in the process of image enhancement at the cost of reducing the score of UIQM. However, it is necessary to sacrifice for the promotion of detailed enhancement of underwater images. Moreover, the visualization results and the quantitative results of the indicators UCIQE and UIQM may not completely match [25], and therefore our experimental results are reasonable.

### 4.5. Evaluation on Underwater Object Detection

**Figure 8:**
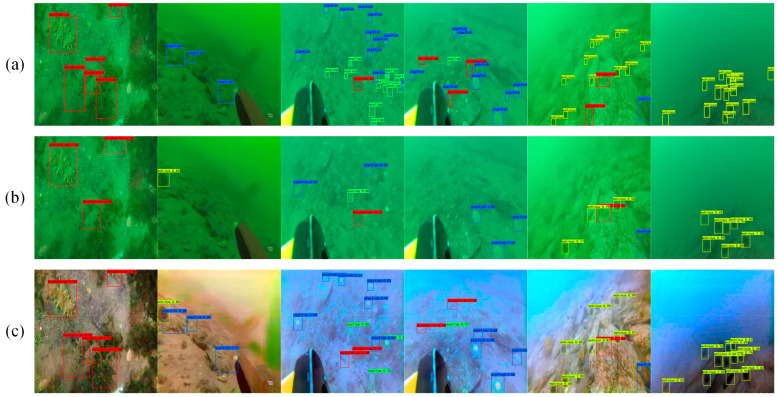
Underwater object detection results before and after enhancement. (**a**) Results with labels. (**b**) Results before enhancement. (**c**) Results after enhancement. Red boxes represent holothurians, blue boxes represent starfishes, yellow boxes represent echinus, and green boxes represent scallops.

The performance of advanced computer vision tasks (such as target detection) for image enhancement can be used as an indicator of image enhancement performance [26]. We ran the YOLO V3 [27] object detection algorithm on the real underwater images and the underwater images enhanced by our method. Figure 8a represents the label, Figure 8b represents the detection results without enhancement, and Figure 8c represents the detection results after the enhancement using our method. The experimental results show that the enhanced underwater image by our method had a better target detection effect than the underwater image before enhancement.

## 5. Conclusions

In underwater image enhancement based on deep learning, it is challenging to collect large sets of underwater data in deep-sea environments. Obtaining the ground truth of the true colors of natural seabed scenes is also an open problem. In addition, most deep learning models fail to retain and transmit details of underwater images fully, and do not fully extract depth features of underwater images, resulting in color imbalance and detail blurriness of recovered images, which limit their applications in practical scenarios. This paper proposes a deep supervised residual dense network for underwater image enhancement that better learns the mapping relationship between clear in-air images and synthetic underwater degraded images. Compared with the other underwater image enhancement method, the proposed method fully retains the details of the content and structure of the original image while defogging and recovering color, obtaining good quantitative and qualitative results.

- Considering that it is difficult to obtain many paired underwater datasets, UWGAN is used for modelling underwater images from in-air images and depths. The generative adversarial network incorporates the process of underwater image formation to generate underwater degraded output images. The synthetic underwater images will be used in the image enhancement network.
- In view of the loss of details in the process of underwater image enhancement, we proposed a deep supervised residual dense network. The network uses residual dense blocks to extract features of different levels and different scales, which can realize feature reuse and reduce loss during feature propagation; moreover, residual path blocks are added to the skip connection between encoder and decoder, which balances the semantic differences between low-level features and high-level features and reduces the loss of spatial information in the down-sampling process.
- A deep supervision mechanism is introduced to construct the accompanying objective function for the hidden layer output of the network, and it guides the network training process and improves the phenomenon that the gradient disappears during the backpropagation of the deep convolutional neural network. This improves the robustness of the network and enables the model to achieve a good enhancement effect on images with different degrees of degradation.

The proposed method is helpful for future research on underwater image enhancement based on deep learning algorithms. Future work will focus on improving the image generation method to produce more realistic underwater degraded images to improve the effectiveness of our DS_RD_Net further.

## Figures and Tables

**Table 1:** Evaluation results of full-reference indicators on synthetic underwater datasets.

Method	MSE ↓	RMSE ↓	PSNR ↑	SSIM ↑
Unet3 [9]	77.7151	8.5030	29.8178	0.9215
Unet4	33.5260	5.4869	33.7995	0.9466
RD-Unet	29.8556	5.1630	34.3432	0.9526
RD_RP-Unet	23.0679	4.5872	35.2685	0.9605
DS_RD_Net	19.3452	4.1534	36.2106	0.9647

**Table 2:** Evaluation results of non-reference indicators on real underwater datasets.

Method	UCIQE ↑	UIQM ↑
Original images	0.4055	2.0483
Unet3 [9]	0.5330	2.8504
Unet4	0.5344	2.6782
RD-Unet	0.5298	2.6806
RD_RP-Unet	0.5376	2.6892
DS_RD_Net	0.5335	2.6653

**Table 3:** Quantitative results evaluation on synthetic underwater datasets by full-reference metrics.

Method	MSE ↓	RMSE ↓	PSNR ↑	SSIM ↑
UCM [1]	2268.064	45.1733	15.5250	0.7998
UDCP [6]	7252.162	82.5960	10.1175	0.4991
UGAN [24]	55.1747	7.1374	31.3839	0.9435
FunieGAN [11]	70.1131	7.6450	31.1095	0.9563
Unet3 [9]	77.7151	8.5030	29.8178	0.9215
DS_RD_Net	19.3452	4.1534	36.2106	0.9647

**Table 4:** Quantitative results evaluation on real underwater datasets by non-reference metrics.

Method	UCIQE ↑	UIQM ↑
Original images	0.4055	2.0483
UCM [1]	0.5730	2.5184
UDCP [6]	0.5099	1.5055
UGAN [24]	0.5570	2.9607
FunieGAN [11]	0.5248	2.8198
Unet3 [9]	0.5330	2.8504
DS_RD_Net	0.5335	2.6653

## Data Availability

Enquiries regarding experimental data should be made by contacting the first author.

## Abbreviations

UCM	Unsupervised color correction method
UDCP	Underwater dark channel prior
UGAN	Underwater generative adversarial network
FunieGAN	Fully convolutional GAN for real-time underwater image enhancement
UWGAN	Underwater GAN for generating realistic underwater images.
Unet3	Unet with three down-sampling
Unet4	Unet with four down-sampling
RD-Unet	Add residual dense blocks based on Unet4
RD_RP-Unet	Add residual path blocks based on RD-Unet
DS_RD_Net	Add a deep supervision based on RD_RP-Unet
MSE	Mean squared error
RMSE	Root-mean-squared error
PSNR	Peak signal to noise ratio
SSIM	Structural similarity index measure
UCIQE	Underwater color image quality evaluation
UIQM	Underwater image quality measurement
